# Hydrocephalus-induced central hypothalamic-pituitary-thyroid axis dysfunction: a conceptual framework linking ventricular distension to behavioral deficit

**DOI:** 10.3389/fendo.2026.1934568

**Published:** 2026-09-22

**Authors:** Emmanuel Garba Sunday

**Affiliations:** 1 Department of Surgery, Division of Neurosurgery, Benue State University Teaching Hospital, Makurdi, Nigeria; 2 Department of Surgery, Federal University Wukari, Wukari, Nigeria

**Keywords:** AQP4, behavioral deficit, central hypothyroidism, computed tomography, glymphatic, hydrocephalus, low-resource settings, Nigeria

## Abstract

**Background:**

Despite successful CSF diversion for pediatric obstructive hydrocephalus, up to 40% of children in Sub-Saharan Africa develop persistent neurodevelopmental delay, growth arrest, and executive dysfunction. Models based on axonal injury or ischemia alone do not fully explain this burden.

**Hypothesis:**

We propose the Hypothalamic Compression-Endocrine-Glymphatic Dysfunction Theory. Third ventricular expansion compresses and shears periventricular hypothalamic neurons, particularly TRH-secreting cells of the paraventricular nucleus. This disrupts pulsatile TRH release and impairs TSH glycosylation. The resulting biologically inactive TSH is detected on routine immunoassays but fails to stimulate the thyroid, causing cryptic central hypothyroidism that standard screening misses.

**Mechanism:**

Thyroid hormone deficiency downregulates and mislocalizes Aquaporin-4 (AQP4) at the neurovascular unit, causing glymphatic stasis and impaired waste clearance. This creates a cycle: ventricular distension suppresses hypothalamic output, inducing central hypothyroidism that compromises microstructural clearance and intracranial compliance, driving neurocognitive deficits despite pressure normalization.

**LMIC relevance:**

In low-resource settings, CT surrogates can stratify risk. Third ventricular width >8 mm plus Evans’ Index >0.30 identifies patients needing targeted free T4 testing. Automated deep-learning CSF segmentation offers scalable diagnosis.

**Conclusion:**

Treating hydrocephalus-induced central hypothyroidism with levothyroxine alongside surgical decompression may improve outcomes. Multicenter trials in Nigeria are needed to validate these CT-endocrine indices.

## 1 Introduction

Obstructive hydrocephalus presents a formidable global neurosurgical burden, with its highest incidence concentrated in Sub-Saharan Africa due to endemic post-infectious and post-hemorrhagic etiologies. While surgical interventions like ventriculoperitoneal shunting and endoscopic third ventriculostomy (ETV) effectively manage intracranial pressure (ICP), a significant subset of pediatric and adult patients exhibit persistent neurodevelopmental deficits, growth failure, and cognitive delays despite normalized CSF dynamics. Traditional paradigms attribute these long-term sequelae to diffuse white matter axonal stretching, periventricular ischemia, and chronic neuroinflammation (1–3).

The secondary metabolic and neuroendocrine consequences of structural ventricular expansion remain less clear. Anatomically, the hypothalamus forms the floor and ventral lateral walls of the third ventricle. Its critical neuroendocrine clusters, particularly the thyrotropin-releasing hormone (TRH)-secreting neurons of the paraventricular nucleus (PVN), reside immediately adjacent to the ependymal lining (4). The vulnerability of these midline structures to mechanical deformation was noted as early as 1919 by Walter Dandy, who observed that ventricular distension could flatten pituitary and hypothalamic tissue (5). Modern histological analyses in animal models confirm severe ependymal disruption, localized gliosis, and neuronal loss within the hypothalamus during chronic ventriculomegaly (6). Furthermore, clinical studies have documented blunted pituitary hormone responses in pediatric cohorts demonstrating significant third ventricular expansion (5–10).

Crucially, emerging paradigms in human-oriented neuroanatomy suggest that the hypothalamus-hypophysis axis does not function in isolation; rather, its structural integrity is deeply intertwined with macro- and microvascular fluid dynamics. Recent frameworks describe an integrated three-interface conceptual model for clearance-plus-immune surveillance in humans, where the parenchymal-CSF boundaries, vascular-interstitial boundaries, and meningeal interfaces act in synergy to regulate regional waste clearance and immune privilege (11). This model has recently been expanded to specifically emphasize the role of the hypothalamus-hypophysis axis as a critical neuroendocrine interface dependent on this clearance network (12). Because the hypothalamus-hypophysis axis rests at a highly specialized neuroendocrine interface characterized by fenestrated capillaries and dense perivascular spaces, it is uniquely dependent on this three-interface network for optimal homeostasis (11, 12). The mechanical stress of third ventricular distension likely disrupts this delicate fluid-immune network, compounding localized tissue stress (11, 12).

This framework targets a specific endocrine and glymphatic consequence of this mechanical process: hydrocephalus-induced central hypothyroidism. This condition is defined biochemically by a subnormal serum free thyroxine (FT4) concentration in the presence of an inappropriately low, normal, or minimally elevated thyroid-stimulating hormone (TSH) level. Because thyroid hormones are essential for axonal myelination, synaptic plasticity, and cerebral metabolic rate, a chronic central hypothyroid state may compound or mimic primary structural brain injury (10, 13). Furthermore, a dangerous feedback loop may exist: recent evidence demonstrates that central hypothyroidism itself impairs global glymphatic flow by altering the polarization of Aquaporin-4 (AQP4) water channels at the neurovascular unit, which drastically compromises convective interstitial fluid drainage (14, 15). Consequently, hydrocephalus-induced hypothalamic suppression could trigger a secondary, systemic hypothyroid state that directly exacerbates glymphatic stasis and delays parenchymal recovery (10, 13–16).

This paper builds a formal conceptual framework linking third ventricular distension to HPT axis suppression and subsequent glymphatic impairment. It addresses diagnostic challenges specific to low-resource environments and advocates for the validation of axial CT indices as low-cost screening tools in neurosurgical units across LMICs, where diagnostic MRI access is severely constrained.

### 1.1 Rationale for CT-based criteria in low-resource settings

The gold standard for assessing hypothalamic deformation is high-resolution MRI with automated volumetrics. However, in Sub-Saharan Africa and other low- and middle-income regions, MRI is unavailable, cost-prohibitive, or has waiting times exceeding 6 months in over 80% of neurosurgical centers (17). In contrast, computed tomography is widely available, rapid, and affordable. Given that an estimated 200 million people in LMICs lack access to MRI, there is an urgent public health need to develop CT-based surrogate criteria for early detection of hypothalamic compression (18). Such a tool would allow for risk stratification and timely endocrine referral in settings where neurosurgical and endocrine resources are severely limited (17–19).

## 2 The conceptual framework

### 2.1 The hypothalamic compression, endocrine, and glymphatic dysfunction theory

The structural architecture of the third ventricle places the hypothalamus at a unique mechanical disadvantage during obstructive hydrocephalus. The paraventricular nucleus (PVN) and the median eminence are highly vascularized structures positioned along the thin periventricular zone. When CSF drainage is impeded—such as in aqueductal stenosis or post-meningitic basilar arachnoiditis—the third ventricle dilates laterally and inferiorly. This expansion subjects the surrounding hypothalamic nuclei to perpendicular compressive forces, tangential shearing vectors, and compromised microvascular perfusion via the long hypophyseal portal vessels (7, 20). Experimental kaolin-induced hydrocephalus models corroborate this vulnerability, demonstrating marked astrocyte activation, microglia infiltration, and selective parvocellular neuronal degeneration within the PVN (7, 20, 21). Standardized protocols for assessing dendritic injury in these hypothalamic nuclei are now available to facilitate future mechanistic validation (22).

Crucially, emerging human-oriented neuroanatomical paradigms indicate that these mechanical stresses do not act in isolation. The hypothalamus-hypophysis axis sits at a highly specialized neuroendocrine junction that is deeply dependent on an integrated three-interface conceptual model for clearance-plus-immune surveillance (11). This network—comprising parenchymal-CSF boundaries, vascular-interstitial boundaries, and meningeal interfaces—regulates regional waste clearance and localized immune privilege (11, 12). The outward bowing of the third ventricular floor and the structural displacement of the mammillary bodies directly disrupt these delicate fluid-interstitial boundaries, severely compromising local homeostatic drainage and amplifying tissue stress (11, 12) (**Figure 1**).

**Figure 1:**
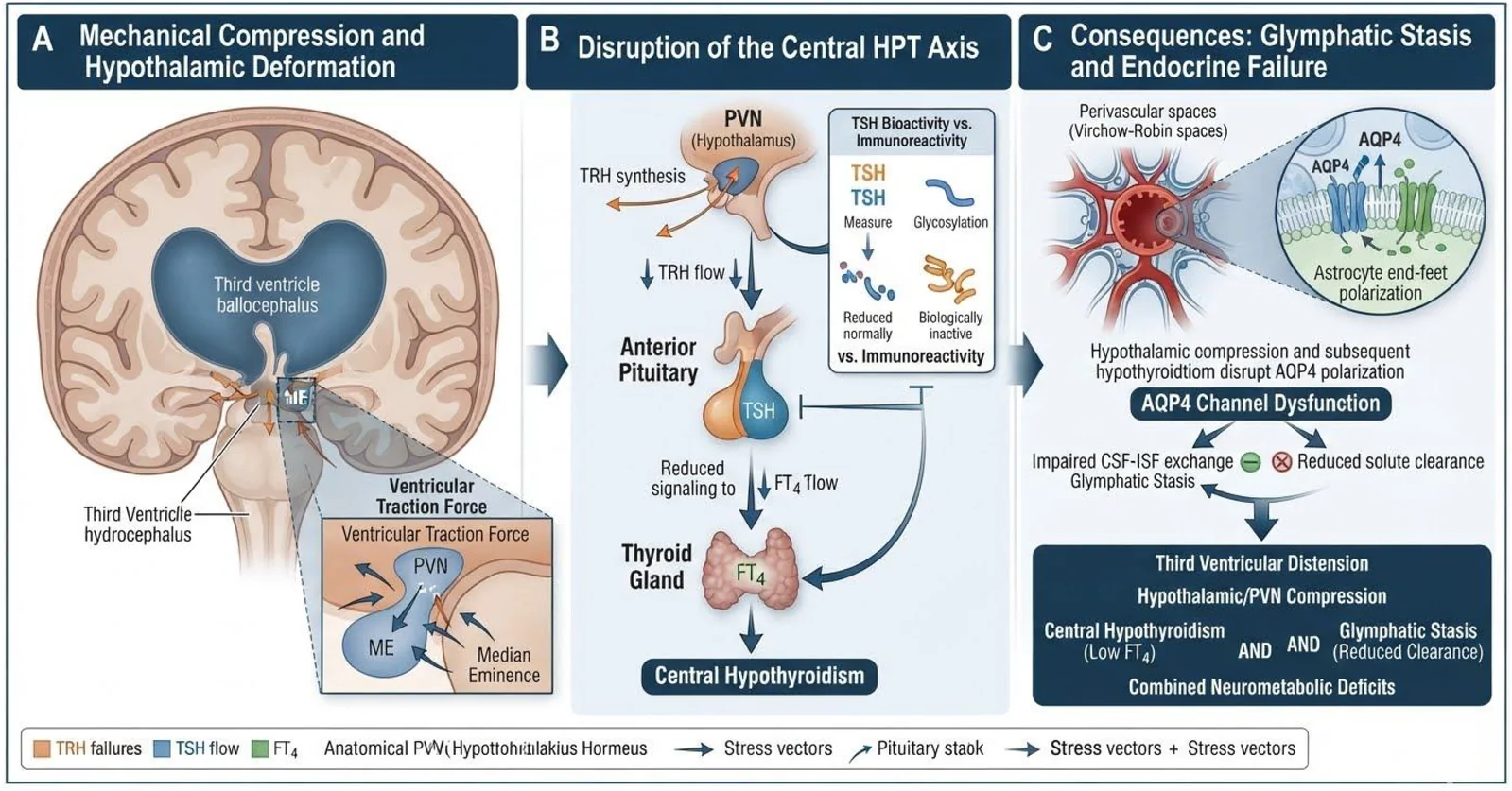
The hypothalamic compression-endocrine-glymphatic dysfunction theory in hydrocephalus. **(A)** Mechanical compression and hypothalamic deformation: obstruction of cerebrospinal fluid (CSF) flow drives progressive distension and lateral expansion of the third ventricle. This macro-structural deformation exerts direct perpendicular compressive forces and tangential shearing vectors (ventricular traction force) across the periventricular zone, targeting the immediate ependymal lining, the paraventricular nucleus (PVN), and the median eminence (ME). **(B)** Disruption of the central HPT axis: Mechanical traction and localized microvascular ischemia stretch the unmyelinated axons of thyrotropin-releasing hormone (TRH)-secreting neurons, impairing TRH synthesis and axoplasmic transport to the hypophyseal portal system. This lack of pulsatile TRH deprives anterior pituitary thyrotropes of mandatory trophic stimulation, disrupting post-translational TSH glycosylation. The resulting immunologically measurable but biologically inactive TSH isoforms fail to stimulate the thyroid gland, triggering central hypothyroidism characterized by depleted free thyroxine (FT_4_) and free triiodothyronine (FT_3_) levels. **(C)** Consequences: glymphatic stasis and endocrine failure: The downstream central hypothyroid state establishes a secondary, self-reinforcing neurometabolic feedback loop. Thyroid hormone deficiency directly disrupts the precise polarization of Aquaporin-4 (AQP4) water channels at the perivascular astrocyte end-feet. This loss of polarization compromises the vascular-interstitial boundary of the newly established three-interface model (comprising parenchymal-CSF, vascular-interstitial, and meningeal interfaces), halting convective glymphatic influx and interstitial fluid (ISF) clearance. The resulting glymphatic stasis reduces global intracranial compliance and exacerbates periventricular fluid accumulation, locking the patient into a vicious cycle of progressive structural deformation, persistent endocrine failure, and permanent behavioral deficits.

This mechanical disruption directly intersects with a multi-layered Endocrine-Metabolic Pathway. First, physical stretching of the unmyelinated axons of TRH-secreting neurons as they course toward the median eminence diminishes hypothalamic TRH release. This deprives anterior pituitary thyrotropes of their primary trophic stimulus. While basal immunoreactive TSH secretion may persist, the lack of pulsatile TRH disrupts the post-translational glycosylation of TSH molecules, culminating in the secretion of TSH isoforms with drastically reduced biological activity (23). Consequently, circulating free thyroxine (FT4) and free triiodothyronine (FT3) levels decline, inducing central hypothyroidism (23).

At the cellular level, this thyroid hormone deficiency triggers a secondary, debilitating glymphatic feedback loop. The glymphatic system was first described as a paravascular pathway facilitating CSF flow and interstitial solute clearance (24). Subsequent work established that glymphatic failure can act as a final common pathway to neurodegeneration (25). Recent evidence demonstrates that a central hypothyroid state directly impairs global glymphatic flow by altering the polarization of Aquaporin-4 (AQP4) water channels at the neurovascular unit (26). This mechanism has been mechanistically linked to reduced CSF influx and interstitial solute clearance in models of endocrine dysfunction (14, 27). This loss of AQP4 polarization drastically compromises convective interstitial fluid drainage and dampens Na^+^/K^+^-ATPase activity within the choroid plexus and brain capillaries (15). Ultimately, hydrocephalus-induced hypothalamic compression establishes a self-reinforcing pathological cycle: mechanical expansion causes central hypothyroidism, which in turn causes glymphatic stasis, worsening interstitial fluid accumulation, and severely reduces overall intracranial compliance (14, 15, 23–27).

While direct human biopsy evidence of PVN injury in hydrocephalus is lacking, preclinical models provide support for this mechanism. In kaolin-induced hydrocephalus in rodents, histological studies demonstrate astrocyte activation, microglial infiltration, and selective neuronal degeneration within hypothalamic nuclei including the PVN (6, 7, 20). To enable future validation of dendritic injury in these regions, we have published a detailed Golgi-Cox protocol optimized for hypothalamic tissue (22). Clinically, studies in pediatric hydrocephalus have documented blunted pituitary hormone responses to stimulation testing (28), and patients with idiopathic normal pressure hydrocephalus frequently exhibit hypopituitarism (29, 30). It is also important to note that the PVN is not solely an HPT regulatory center. It is the primary site of oxytocin synthesis and has extensive connections with dopaminergic pathways (31, 32). While this framework focuses on the HPT axis for its clinical testability in LMICs, we acknowledge that co-disruption of oxytocin and dopamine circuits may represent additional contributors to the behavioral phenotype and should be explored in future work (31–33).

### 2.2 Limitations of the conceptual framework

While pathophysiologically plausible, several confounding factors and structural limitations must be acknowledged within this working model. First, this conceptual framework simplifies a highly complex physiological milieu. In clinical scenarios, significant neuroinflammatory cascades, oxidative stress, and periventricular ischemia occur concurrently with mechanical ventricular expansion, making it difficult to isolate pure mechanical compression of the hypothalamus from global ischemic changes (34, 35). Furthermore, mapping the precise real-time dynamics of the newly integrated three-interface network and localized Aquaporin-4 (AQP4) polarization remains a profound challenge in human subjects (11). Experimental kaolin-induced hydrocephalus models, while informative for cellular tracking, induce widespread, multifocal intracranial changes that may not perfectly mirror the chronic, isolated third ventricular expansion seen in human clinical cohorts (20).

This complexity is further highlighted by substantial clinical heterogeneity, as some patients present with profound central hypothyroidism and severe glymphatic stasis, while others maintain completely normal thyroid panels and fluid clearance dynamics despite matching degrees of macrocephaly and ventriculomegaly. Finally, from a diagnostic standpoint, axial CT dimensions serve only as indirect estimates of macro-structural tissue tension (36). They lack the cellular and structural precision offered by advanced MRI deformation grading scales required to accurately quantify interstitial fluid boundaries or localized neurovascular unit disruption (36). Consequently, this framework is presented as a dynamic working model designed to guide prospective multi-center verification and targeted biomarker exploration rather than as an established clinical fact. Future studies should also investigate the role of co-disrupted oxytocinergic and dopaminergic signaling from the PVN as additional drivers of behavioral and social deficits in hydrocephalus (33).

## 3 The HPT axis in brain injury: physiology, clinical features, and diagnostic challenges

### 3.1 Clinical features of hypothalamic dysfunction and behavioral correlates

Hydrocephalus-induced hypothalamic injury can present with a constellation of endocrine and behavioral symptoms that are often attributed to the hydrocephalus itself. Clinicians should screen for growth failure in children, temperature dysregulation, sleep-wake cycle disturbances, appetite and weight changes, and disorders of puberty. Biochemically, the hallmark of central hypothyroidism is a low free T4 level with an inappropriately normal or low TSH (16). A full pituitary panel including morning cortisol, LH, FSH, and IGF-1 is recommended to rule out panhypopituitarism and to differentiate this from non-thyroidal illness syndrome. Mechanistically, the link to behavioral deficits is direct. Thyroid hormone is essential for neuronal myelination, synaptogenesis, and regulation of cerebral metabolic rate during development (10, 13). A chronic state of central hypothyroidism therefore provides a plausible endocrine basis for the executive dysfunction, slowed processing speed, attention deficits, and developmental delay observed in children with hydrocephalus, independent of direct white matter injury (37–39).

### 3.2 Physiology and diagnostic challenges

The homeostatic regulation of the HPT axis depends on the precise, pulsatile secretion of hypothalamic TRH into the hypophyseal portal system (4). This triggers the synthesis and release of pituitary TSH, which drives the thyroid gland to produce T4 and T3 (9). Central hypothyroidism occurs when structural or functional defects at the level of the hypothalamus or pituitary blunt this cascade (4, 9).

A major diagnostic hurdle in neurosurgical practice is differentiating true central hypothyroidism from Non-Thyroidal Illness Syndrome (NTIS), also known as euthyroid sick syndrome. During acute systemic stress, severe infection, or severe malnutrition—all common clinical presentations in LMIC neurosurgical wards—peripheral deiodinase activity is altered (40). This suppresses serum T3 levels and, in prolonged illness, reduces both TRH expression and TSH pulse amplitude (40). This systemic down-regulation mimics central hypothyroidism without requiring an underlying mechanical or structural injury to the hypothalamus (40, 41). Less well-known aspects of central hypothyroidism include diagnostic pitfalls related to biologically inactive TSH isoforms (41).

Compounding this challenge is the fact that current neuroendocrine literature focuses primarily on intrinsic pituitary adenomas, craniopharyngiomas, or traumatic brain injury (TBI). Clinical data detailing the pre- and post-operative longitudinal profiles of TRH/TSH dynamics specifically in the context of chronic mechanical ventricular expansion remain limited, particularly within the unique demographic and clinical landscape of Sub-Saharan Africa (42).

## 4 Clinical evidence and geographic gaps

### 4.1 Overview of current literature

Retrospective clinical reviews link pediatric hydrocephalus to an array of endocrine abnormalities, including somatic growth failure, precocious or delayed puberty, and secondary adrenal or thyroid insufficiency (28). In adult cohorts presenting with idiopathic normal pressure hydrocephalus (iNPH), objective pituitary-hypothalamic dysfunction has been documented, with a subset of these endocrine deficits reversing following successful surgical shunting (29). However, these existing datasets are largely single-center retrospective series that lack standardized imaging metrics or uniform laboratory collection timelines (28, 29).

### 4.2 The sub-Saharan African pathological context

Crucially, there is a total absence of peer-reviewed neuroendocrine data for hydrocephalus patients from Sub-Saharan Africa. In Nigeria, the clinical picture of hydrocephalus is shaped by delayed presentation, prolonged periods of elevated ICP, and a high prevalence of post-infectious etiologies like neonatal sepsis and tuberculous or meningococcal meningitis (2, 42). The intense inflammatory scarring that occurs within the optico-chiasmatic and interpeduncular cisterns in post-infectious hydrocephalus likely causes direct ischemic and fibrotic changes to the infundibular stalk (43). This tissue damage compounds the mechanical stress caused by ventricular enlargement (44). Because of these distinct pathological differences, neuroendocrine conclusions drawn from early-presentation congenital hydrocephalus cohorts in high-income countries cannot be directly generalized to the Nigerian clinical context (2, 42–44).

## 5 Central hypothyroidism in hydrocephalus: prevalence and mechanism

### 5.1 Prevalence and biochemical basis

Available international data indicate that the prevalence of central hypothyroidism in pediatric hydrocephalus cohorts ranges from 8% to 35%, depending on patient age, the underlying etiology, and the stringency of the diagnostic criteria applied (8). The biochemical mechanism centers on the loss of pulsatile TRH signaling, which alters the terminal sialylation and sulfation of TSH oligosaccharide chains within the anterior pituitary (23). This structural change produces a TSH molecule that remains fully detectable by conventional, automated sandwich immunoassays but lacks the stereochemical configuration required to bind and activate the follicular TSH receptor on the thyroid gland (23, 41).

As a result, a clinician relying solely on a routine TSH screening strategy will miss the diagnosis. The assay returns a value within the “normal” reference range, masking an underlying state of tissue-level hypothyroidism that can be uncovered only by checking a concurrent FT4 level (23). A unique example is neonatal detection of central hypothyroidism due to transient axis suppression from elevated intracranial pressure (45). Currently, no published studies in Sub-Saharan Africa have evaluated hydrocephalus cohorts using these specific neuroendocrine parameters alongside matching neuroimaging scans (19). Furthermore, while validated qualitative MRI scoring systems exist to grade hypothalamic deformation in conditions like craniopharyngioma-induced obesity (19, 46), no standardized imaging scale has been universally adapted or validated for hydrocephalus.

### 5.2 Linking HPT axis disruption to behavioral deficits

The term “behavioral deficit” in hydrocephalus encompasses executive dysfunction, impaired attention, slowed processing speed, poor working memory, and developmental delay. While these deficits are traditionally attributed to periventricular white matter injury and increased intracranial pressure, we propose an additional endocrine mechanism. Thyroid hormone is non-negotiable for normal brain development (10). It regulates neuronal migration, dendritic arborization, synaptogenesis, and oligodendrocyte-mediated myelination, particularly in the frontal-subcortical circuits that underlie executive function (13).

A chronic central hypothyroid state resulting from hypothalamic compression and impaired TRH/TSH secretion therefore provides a plausible biological substrate for the cognitive and behavioral phenotype seen in children with hydrocephalus (10, 13). Importantly, this mechanism may persist even after successful CSF diversion if the central HPT axis is not screened and treated. Early detection and thyroxine replacement could thus represent a modifiable target to improve neurodevelopmental outcomes in hydrocephalus, especially in LMICs where rehabilitation services are limited. Thyroid hormone status has also been directly correlated with health-related quality of life in NPH patients, with normalization after shunting associated with improved outcomes (47).

## 6 Imaging surrogates: CT vs. MRI in resource-limited settings

While high-resolution MRI remains the gold standard for mapping the micro-structural deformation of the hypothalamus and quantifying disruptions to perivascular spaces, its clinical utility is severely constrained in low- and middle-income countries (LMICs) due to high costs and limited availability (17). The burden of neuroimaging access in Sub-Saharan Africa specifically highlights this gap (17). In resource-constrained environments like Sub-Saharan Africa, where advanced neuroimaging is frequently unavailable or introduces prohibitive delays, a scalable and cost-effective screening surrogate is urgently required (12, 17). This framework proposes utilizing routine, widely accessible axial computed tomography (CT) metrics to identify patients at high risk for hydrocephalus-induced central hypothyroidism and secondary glymphatic failure (12, 17) (**Figure 2**).

**Figure 2:**
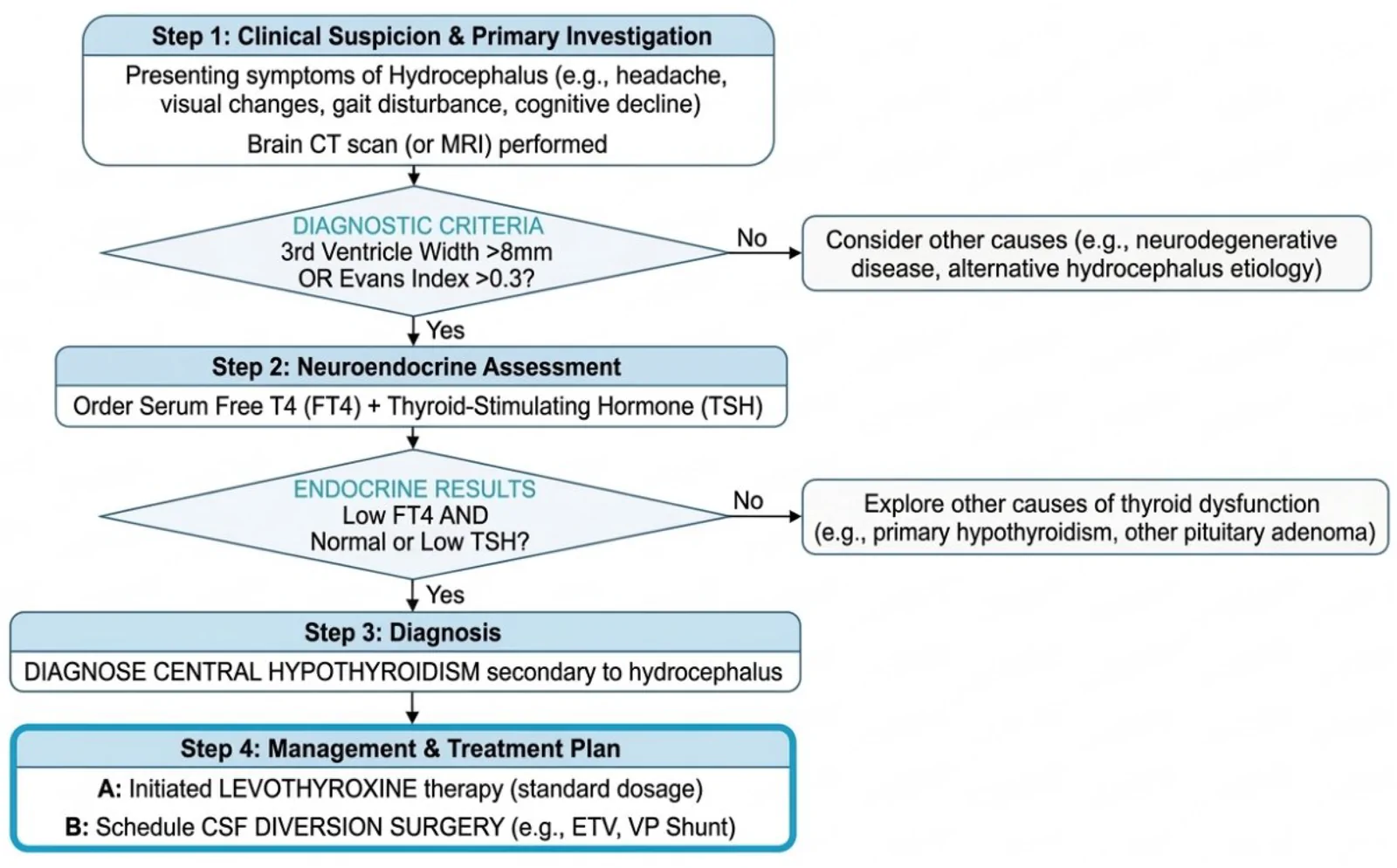
Proposed CT-guided screening algorithm for central hypothyroidism and glymphatic risk triage in LMICs. Step 1 initiates screening based on classic hydrocephalus symptoms, explicitly incorporating behavioral and developmental delays alongside standard neurological deficits. Step 2 utilizes accessible axial CT structural surrogates (third ventricular width >8 mm or Evans’ index >0.3) to identify severe third ventricular floor expansion capable of disrupting local tissue boundaries. Step 3 targets the biochemical pitfall of central hypothyroidism by mandating concurrent FT_4_ and TSH testing, ensuring that biologically inactive TSH isoforms do not mask underlying tissue-level hypothyroid states. Step 4 outlines a synergistic, two-pronged treatment paradigm combining metabolic optimization (levothyroxine therapy) with mechanical decompression (CSF diversion surgery) to restore both endocrine balance and AQP4-mediated glymphatic clearance.

As outlined in the clinical algorithm, the screening process is triggered by primary clinical suspicion based on classic macro-structural hydrocephalus presentations, including headaches, visual changes, gait disturbances, and persistent behavioral or developmental deficits. When an axial CT scan is performed, two reproducible macro-structural metrics serve as proxies for microscopic tissue stress: a third ventricular width greater than 8 mm and an elevated Evans’ index greater than 0.3 (36). A third ventricular diameter exceeding 8 mm indicates significant lateral and inferior ballooning of the third ventricular floor (36). This specific structural expansion acts as a reliable surrogate for the perpendicular compression and tangential shearing forces that disrupt the paraventricular nucleus (PVN), the median eminence, and the surrounding vascular-interstitial boundaries (36).

Patients meeting these structural CT criteria are immediately triaged for a targeted neuroendocrine panel consisting of serum free thyroxine FT4 and thyroid-stimulating hormone (TSH). If the lab results reveal a low FT4 concentration paired with an inappropriately normal or low TSH level, a diagnosis of secondary central hypothyroidism is confirmed (23). This prompt identification bypasses the standard diagnostic pitfall where automated assays mistake biologically inactive TSH isoforms for normal endocrine function (23, 41). Confirmed cases are managed using a two-pronged therapeutic strategy: the initiation of weight-adjusted levothyroxine therapy to restore systemic thyroid hormone levels, alongside the scheduling of definitive surgical CSF diversion (e.g., endoscopic third ventriculostomy or ventriculoperitoneal shunting) (14, 15, 23).

Restoring the thyroid profile pre-operatively or immediately post-operatively is critical; correcting the central hypothyroid state re-establishes normal Aquaporin-4 (AQP4) water channel polarization at the neurovascular unit, thereby optimizing global glymphatic clearance, improving post-operative intracranial compliance, and accelerating long-term behavioral recovery (14, 15, 47). This aligns with longitudinal data showing that thyroid hormone normalization following VP shunt is associated with improved health-related quality of life in NPH (47).

## 7 Gaps and research agenda

### 7.1 Identification of current gaps

To transition this conceptual framework into evidence-based clinical protocols, four primary research gaps must be addressed:

- Lack of Prospective Data: There are no prospective studies directly correlating linear CT measurements with matched FT4/TSH values before and after surgical intervention.
- Absence of Regional Baselines: There is a total lack of clinical neuroendocrine data from Nigerian hydrocephalus cohorts to establish regional baseline characteristics.
- No Direct Metric Comparisons: Head-to-head statistical comparisons have not been performed to determine whether third ventricular width, Evans’ index, or the frontal horn ratio best predicts endocrine failure.
- Diagnostic Confounding: Improved clinical strategies are needed to clearly differentiate true mechanical central hypothyroidism from systemic NTIS in acutely ill neurosurgical patients (40).

### 7.2 Proposed research priorities

We propose a structured research agenda to address these gaps across collaborating Nigerian institutions, including Benue State University Teaching Hospital (BSUTH), Makurdi, and partner tertiary networks:

#### 7.2.1 Prospective multi-center cohort study

Implement a standardized protocol to collect pre-operative and longitudinal post-operative (3–6 months) serum FT4 and TSH levels alongside routine brain CT scans.

#### 7.2.2 Validation of imaging indices

Calculate the sensitivity, specificity, and receiver operating characteristic (ROC) curves of third ventricular width (>8 mm) against biochemically proven central hypothyroidism.

#### 7.2.3 Adaptation of morphometric scores

Adapt existing MRI-based hypothalamic injury grading scales into simplified, CT-compatible linear assessment tools.

#### 7.2.4 Feasibility and cost-benefit analysis

Assess the clinical and economic feasibility of adding routine, targeted FT4/TSH testing into pre-operative workups for patients flagged by high-risk CT metrics.

### 7.3 Future directions in volumetric assessment

Future Direction: Validation of automated deep learning tools for CSF volumetrics may provide an objective, scalable alternative to manual CT indices for predicting endocrine and behavioral outcomes in LMICs (15). Modern AI architectures that merge Dense-Net and U-Net configurations demonstrate excellent accuracy, segmenting intracranial compartments from neonatal brain tissue in just 0.8 seconds per scan while outputting high Dice scores (95.7% for CSF; 96.4% for brain parenchyma) (15). Migrating these lightweight automated networks into serverless cloud frameworks or smartphone imaging applications across Sub-Saharan Africa could provide an objective, macro-structural volumetric baseline to optimize diagnostic precision where manual parsing errors introduce variance (15).

## 8 Implications for practice and conclusion

Validating these linear CT ventricular metrics could provide neurosurgeons in resource-limited settings with a clear, low-cost screening trigger. When a pre-operative brain CT reveals severe third ventricular expansion (>8 mm), clinicians can proactively order targeted FT4$= and TSH panels, even when MRI or formal endocrinology consults are unavailable.

Identifying and treating an underlying central hypothyroid state through affordable levothyroxine replacement could address a hidden, reversible cause of post-operative developmental delay. This simple intervention has the potential to maximize cognitive and physical recovery long after CSF diversion surgery has resolved the primary structural issues of hydrocephalus. Evidence links post-operative thyroid status to patient-reported quality of life (47).

In conclusion, mechanical compression of the third ventricular floor represents a plausible but underdiagnosed driver of central HPT axis disruption in hydrocephalus (29, 48, 49). This conceptual framework provides an actionable, testable model tailored for LMIC neurosurgical practice. It highlights the value of using routine CT metrics to guide metabolic screening, offering an opportunity to optimize long-term neurodevelopmental outcomes for patients across Sub-Saharan Africa (29, 48).
